# Objective Assessment of Financial Decision-Making With a Simulated Online Money Management Task in Older Adults: Protocol for a Prospective Observational Study

**DOI:** 10.2196/82488

**Published:** 2026-04-20

**Authors:** Preeti Sunderaraman, Madison Bouchard-Liporto, Zachary Kunicki, Edward D Huey, Stephanie Cosentino

**Affiliations:** 1 Department of Psychiatry and Human Behavior, Alpert Medical School, Brown University, 222 Richmond Street, Providence, RI, 02903, United States; 2 Memory and Aging Program, Butler Hospital, 345 Blackstone Boulevard, Providence, RI, 02906, United States, 1 (401) 455-3000 ext 21119; 3 Department of Neurology, Chobanian & Avedisian School of Medicine, Boston University, Boston, MA, United States; 4 Taub Institute for Research on Alzheimer’s Disease and the Aging Brain, Columbia University Irving Medical Center, New York, NY, United States; 5 The Gertrude H. Sergievsky Center, College of Physicians and Surgeons, Columbia University Irving Medical Center, New York, NY, United States; 6 Department of Neurology, College of Physicians and Surgeons, Columbia University and the New York Presbyterian Hospital, New York, NY, United States

**Keywords:** financial management, decision-making, neuropsychology, technology, cognition, functional status, older adults

## Abstract

**Background:**

Technology-enabled tasks to conduct financial transactions are ubiquitous around the world. In a recent survey, about 75% of the respondents endorsed the use of technology to perform financial activities such as reviewing bank statements and keeping track of money spent. However, assessment of financial decision-making (FDM) is limited by tasks that use traditional paper-and-pencil methods or by relying on self or informant reports. Furthermore, such tools have weak psychometric properties, are prone to biases, and are at times limited in scope. Thus, there is an urgent need to develop modern, technology-based tools that have strong psychometric properties and that can assess FDM comprehensively and accurately.

**Objective:**

This study aimed to develop and establish the psychometric properties of a novel, simulated Online Money Management (OMM) credit card task. Based on existing gaps identified in the literature, this task relied on objective measurement, assessed multiple dimensions within a single task, and mimicked a real-world task to bridge the gap between a controlled, clinical setting and real-world functioning.

**Methods:**

This was a prospective cohort study that enrolled cognitively healthy older adults. This study was funded by the National Institutes of Health. Various recruitment sites were involved, which allowed for the recruitment of older adults across the United States. The OMM task was developed in collaboration with an interdisciplinary team of computer scientists, economists, psychologists, and geriatricians. The tasks consist of both online and offline components, with subcomponents examining the ability to navigate, basic and complex credit card literacy, and statement monitoring. Data about participants’ perception of their financial abilities and a self-report survey on financial exploitation were collected. The test battery consisted of an array of cognitive, financial, and psychosocial tasks. Participants provided written informed consent, and all procedures received institutional review board approval.

**Results:**

Data collection began in September 2019, and enrollment stopped in July 2025. A total of 272 participants completed the baseline visit, while 147 completed the longitudinal follow-up visit. Data analysis is underway as of August 2025, and results are expected to be published in 2026.

**Conclusions:**

Rigorous standards have been deployed for developing this novel OMM credit card task. If the measurement properties of the task are found adequate, the OMM task can be used to assess FDM in clinical evaluations for early detection and prevention or mitigation of financial mismanagement.

## Introduction

Older adults have faced economic hardship in part due to the COVID-19 pandemic and the rising rates of inflation [1]. The level of debt incurred by this group has tripled [2]. According to the National Council on Aging, 15 million households for older adults aged 60 years and older have no assets with a median income of US $18,000 in 2020 [3]. Additionally, age-related cognitive decline, including that associated with Alzheimer disease (AD), increases susceptibility to poor decision-making [4,5], with older adults exhibiting compromised financial decision-making (FDM) in areas such as investment choices [6,7], bankruptcy claims [8], and credit card behaviors [4]. Additionally, older adults are exploited by family members, friends, and strangers more frequently than younger adults [9-13]. Compromised self-awareness can further impact FDM [14,15]. It is therefore critical to be able to identify deteriorating FDM abilities objectively (without relying on self or informant report) in older adults to prevent potentially devastating financial losses [12,16-18]. Early identification can also empower older adults and their care partners to make choices with the goal of preserving wealth.

Use of technology to conduct financial activities has become ubiquitous, even among older adults [19]. In a recent study specifically designed to assess financial habits, the majority of respondents (≈75%) endorsed the use of technology (eg, online services accessed via a computer) to perform financial activities such as reviewing bank statements and keeping track of money spent [19]. The youngest baby boomers, born in 1964 and currently aged 59 years, are expected to be tech-savvy in similar ways to younger cohorts [20,21]. Questions pertaining to online financial management abilities will become increasingly relevant as the number of baby boomers continues to increase precipitously. Simultaneously, there will be a corresponding increase in age-related disorders such as dementia [8,20,22,23]. Timely and modern ways of assessing FDM abilities in vulnerable individuals are critical to preventing and mitigating catastrophic financial loss.

However, there is a lack of instrumentation to evaluate one’s ability to manage finances using modern approaches [24]. Existing instruments are limited because they rely on subjective reports (prone to biases) [25,26] or use traditional, paper-and-pencil methods (eg, checkbook management), which are less relevant to modern, electronic financial management [24,27,28]. Using the COSMIN (Consensus-Based Standards for the Selection of Health Measurement Instruments), a recent review concluded that available instruments had weak psychometric properties [27]. The US Social Security Administration found that current approaches to FDM assessment (eg, clinical interview) were grossly inadequate in predicting real-world FDM and underscored the need to develop sound tools [24,27]. Therefore, the proposed study aimed to develop a simulated Online Money Management (OMM) credit card task to assess FDM and financial awareness in typical cognitive aging.

**Figure 1.**
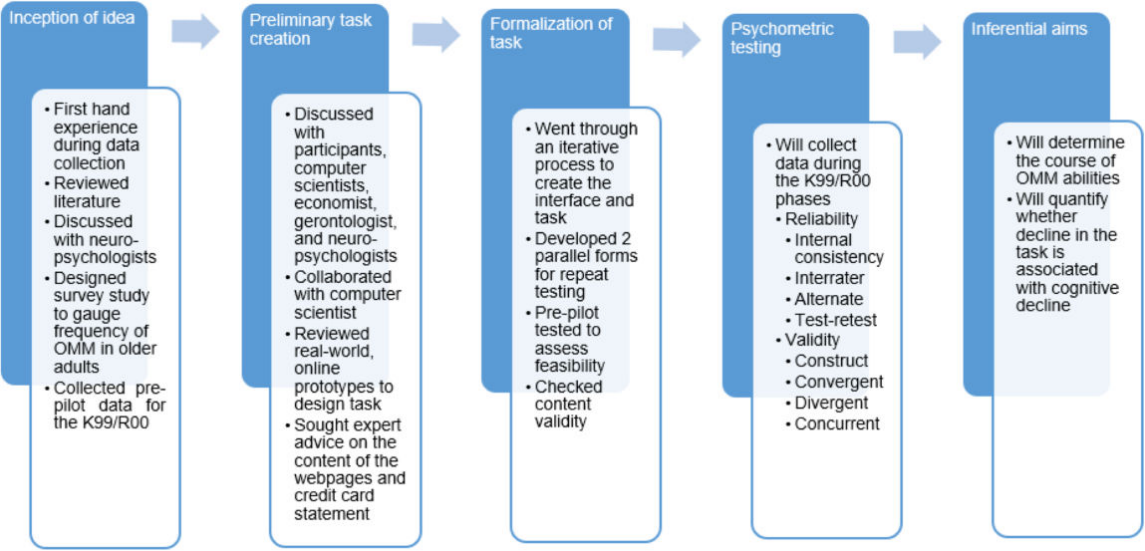
Flowchart depicting the process leading up to the development of the OMM credit card task. OMM: Online Money Management.

The process leading to the development of the OMM task and the K99/R00 project is depicted in Figure 1. Based on survey data collected during the principal investigator’s (PI)’s National Institutes of Health (NIH)–funded F32 project, 61% of baby boomers reported using OMM methods, and 67% reported that using technology was a change from how they previously performed financial transactions[19]. Analysis for different types of financial habits revealed that 65% of older adults were using technology to pay bills, and 80% to check their credit card and bank statements[19]. Conceptual models of FDM have not yet taken OMM methods into account—this is understandable, as the financial landscape has undergone dramatic changes only over the past decade, and some of the influential FDM models were developed before the digital technology proliferation. The 3 leading conceptual models that served as the basis for the OMM task are detailed in the following paragraphs.

The person-centered model [25] focuses on a single impending financial transaction using a person-centered approach that views the individual as a whole with unique strengths, weaknesses, and experiences. It seeks to merge a person’s decisional abilities (choice, understanding, appreciation, and reasoning) with financial awareness of the situation to detect risk of exploitation or fraud. The Lichtenberg Financial Decision-Making Rating Scale [25] and Lichtenberg Financial Decision-Making Rating Screening Scale [28] have been derived from this model, and these instruments are still undergoing psychometric validation [13,28-30]. Though comprehensive in many respects, a limitation of these instruments is that they are based on self-reports, and information gleaned is difficult to objectively verify and/or may be invalid in those with impaired self-awareness or memory problems.

From the clinical-cognitive model [31-34], primarily based on a clinical perspective and borrowing concepts from cognitive psychology, this model conceptualizes financial skills to range from basic (identify and count coins or currency) to complex (make investment decisions) that are necessary for independent functioning. Per the model, financial capability comprises performance, procedural, knowledge or declarative, and judgment-based dimensions [35]. Several instruments, such as the Financial Capacity Instrument – Short Form (FCI-SF), have been derived from this model [35,36]. This model acknowledges that the 3 FDM dimensions may interact, for example, creating a will or investing in a bond requires a combination of all 3 abilities [31]. Thus, financial assessment should ideally include an examination of all 3 dimensions (declarative, procedural, and judgment-based) within a single task. A recent exploratory factor analysis identified 4 factors in the FCI-SF: basic monetary knowledge and calculation skills (eg, calculate a tip), financial judgment (eg, identify mail fraud), financial conceptual knowledge (eg, define debt), and financial procedural knowledge (eg, write a check) [37]. While this model highlights the role of performance-based financial assessment, a limitation of the instrument is that it favors breadth over depth in that it does not fully explore any single dimension of FDM, such as conceptual knowledge and its manipulation.

From the Institute of Medicine Model [24], it and the National Academy of Sciences convened an expert panel for the US Social Security Administration to conduct an exhaustive review of literature and develop a conceptual model of financial capability. Building on the existing models described above, the newly proposed model distinguished between financial competence (typically assessed in a controlled, clinical setting) vs financial performance (real-world functioning). They further divided financial competence into knowledge and judgment and emphasized that to understand real-world functioning, it is important to consider environmental and personal factors (together referred to as “contextual” factors). Personal factors refer to concepts intrinsic to the person (eg, religion, substance addiction, and mental health issues), and environmental factors involve exogenous facets (eg, support system, access to autopay, and access to bank account and financial products). These factors can either impede or facilitate real-world financial performance. The committee concluded that a gap exists between financial competence and financial performance. The OMM task attempts to bridge this gap by embedding experimental, contextual manipulations of specific FDM components (eg, financial knowledge and financial awareness) within a simulated real-world task.

Currently, only a handful of online tools exist to examine FDM. These include the functional skills and assessment training [38], the banking app [39], and the web-based evaluation of banking skills [40]. However, none of these tools comprehensively assesses credit card management. The OMM credit card task will begin to fill in the lacunae in existing FDM models and instruments by (1) measuring abilities directly and objectively, (2) assessing multiple clinical or cognitive dimensions within a single FDM task, and (3) mimicking a real-world task to bridge the gap between a controlled, clinical setting and real-world functioning. The OMM task is developed to make FDM assessment more accurate. It can be used by any trained and qualified professional, including social workers, occupational therapists, physicians, and psychologists. Ultimately, this task will aid in the detection and prevention or mitigation of financial mismanagement and loss experienced by older adults.

The K99/R00 study had four specific aims: (1) to evaluate the reliability, factor structure, and validity of the OMM credit card task in cognitively healthy older adults; (2) to determine the natural course of overall OMM and specific OMM components over a span of 2 years; (3) to examine differences in OMM across cognitively healthy older adults and those with clinical AD; and (4) to examine the nature and strength of the association between financial exploitation and the OMM task in pathological aging.

## Methods

### Study Design

This was a prospective cohort study enrolling a convenience sample of older adults. This study was funded by the NIH, and the grant review summary statement is included in Peer Review Report 1.

### Study Population and Setting

Cognitively healthy older adults and 50 participants with a clinical diagnosis of AD were expected to complete the OMM task. They were to be recruited from the Alzheimer’s Disease Research Center (ADRC) at Columbia University Medical Center (K99 phase) and Boston University School of Medicine (R00 phase). Additional sources of recruitment included the Aging and Dementia Clinic in the Department of Neurology at Columbia University Irving Medical Center (K99 phase) and the Saint Elizabeth’s Hospital in Brighton, MA; Boston Medical Center (BMC) in Boston, MA; and from ResearchMatch, which allowed for recruitment of older adults across the United States (R00 phase).

### Ethical Considerations

This study was approved by the Columbia University Medical Center Institutional Review Board (IRB; K99 phase; protocol number 19‐00364) and by the Boston University IRB (R00 phase; protocol number 66322). Across these institutes, the IRBs determined that this research involves no greater than minimal risk. Study participants provided written informed consent. Participant data are stored in a secure manner using encrypted and password-protected devices and software. Participants were paid US $50 via gift card upon completion of every study time interval they participated in (baseline, 3-month follow, and longitudinal follow-up).

### Eligibility Criteria

Study-specific inclusion and exclusion criteria are detailed in Textbox 1. Aside from the clause about the absence of a clinical diagnosis and independent living, all other criteria apply to those recruited with clinical AD.

Textbox 1. **Inclusion and exclusion criteria for Aims 1 and 2.**

Inclusion criteria

- Living independently in the community.
- Able to read and understand the consent form.
- Native English speaker.
- Reading level above fifth grade.
- Comfortable navigating a computer.
- Manages bank statements or credit card accounts online.

Exclusion criteria

- Montreal Cognitive Assessment score ≤26.
- Diagnosis of neurological disorder.
- Diagnosis of past or current psychotic disorder.
- Current or recent (past 5 years) history of major depressive disorder, bipolar disorder, or anxiety disorder.
- Recent use (past month) of recreational drugs.
- Diagnosed with a learning disability, dyslexia, or attention deficit hyperactivity disorder.

Due to the COVID-19 pandemic, this study procedure had to be initially ramped down and then adapted to use remote testing procedures. The following is a list of study procedures that were changed:

1. The K99 aims could not be fully examined in the proposed timeline of two years due to the circumstances posed by the pandemic. Therefore, baseline data collection on cognitively healthy older adults continued in the R00 phase.
2. During the K99 phase, the PI proposed collecting data on two alternate forms of the OMM credit card statement. Although an alternate version of the OMM credit card task was developed, data collection did not begin due to difficulty recruiting participants online for this study’s sessions during the COVID-19 pandemic.
3. Referral rates for cognitively healthy older adults were low. Consequently, individuals were recruited from the community using flyers posted at public locations (eg, cafes) and through flyers distributed on online websites such as Research Match.
4. Despite recruiting through multiple sources, referrals of patients with AD were infrequent. Therefore, aim 3 of this study comparing the performance of cognitively healthy participants with those having clinical AD was not conducted.

### Study Assessments and Outcome Measures

**Figure 2.**
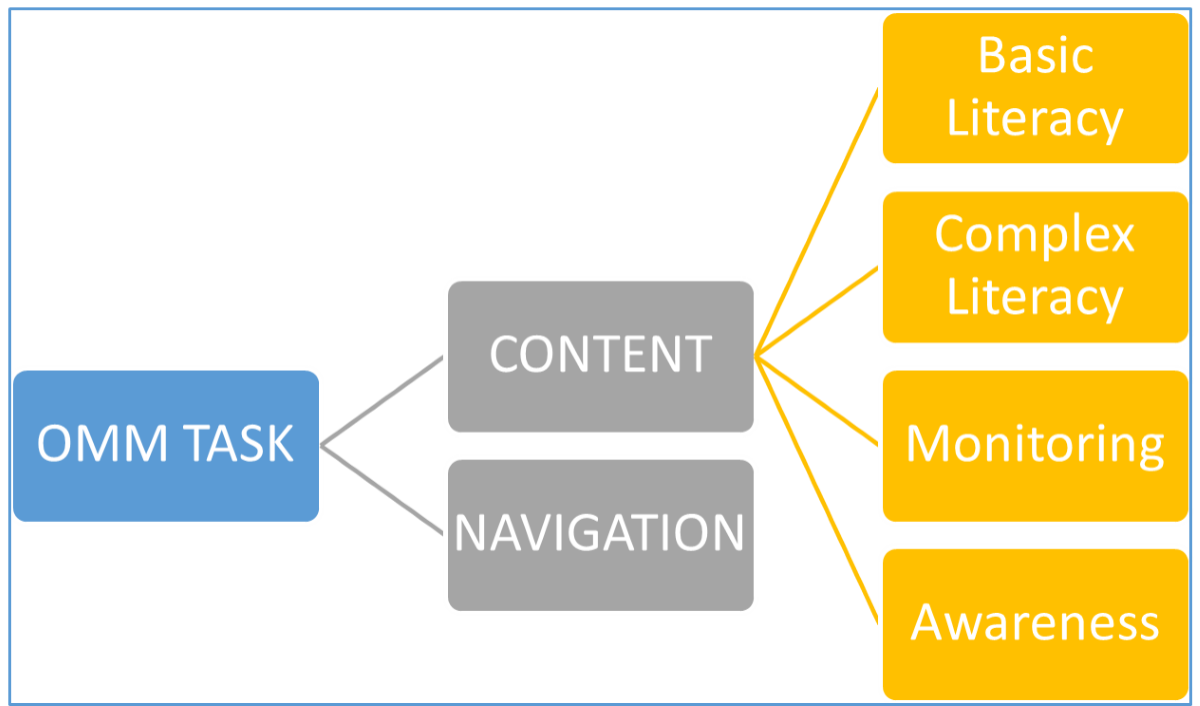
Composition of the OMM task*.* There are 2 broad components of the task: Content and Navigation, which are theoretically independent. The Content component consists of 4 theoretically related subcomponents. OMM: Online Money Management.

**Figure 3.**
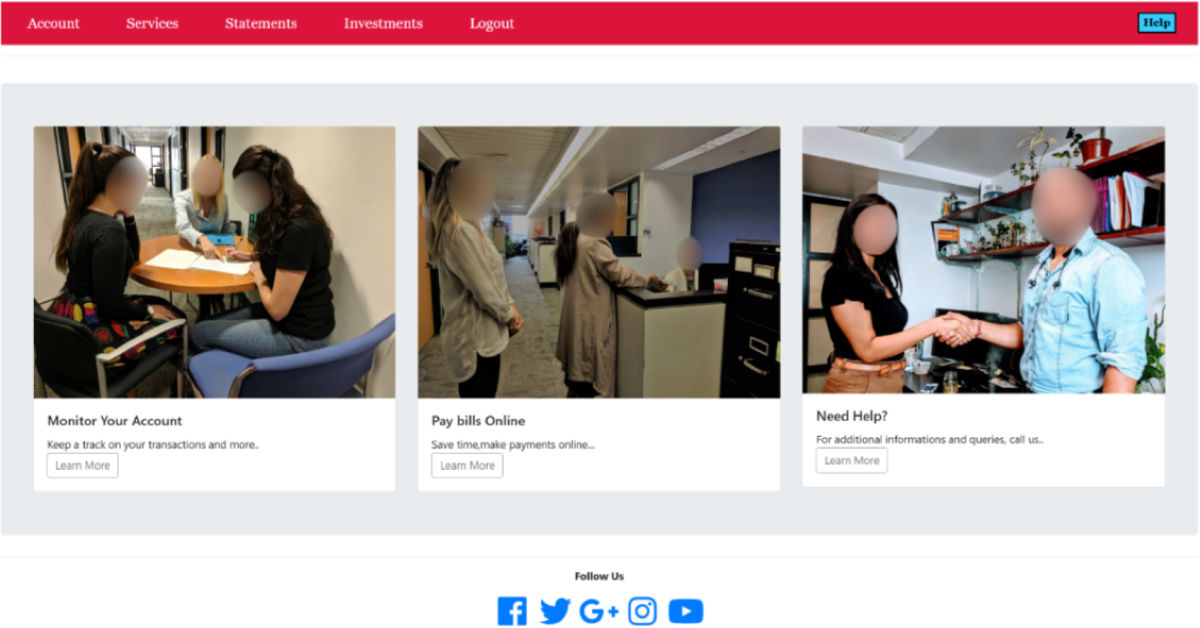
Screenshot of the simulated accounts activity web page.

**Figure 4.**
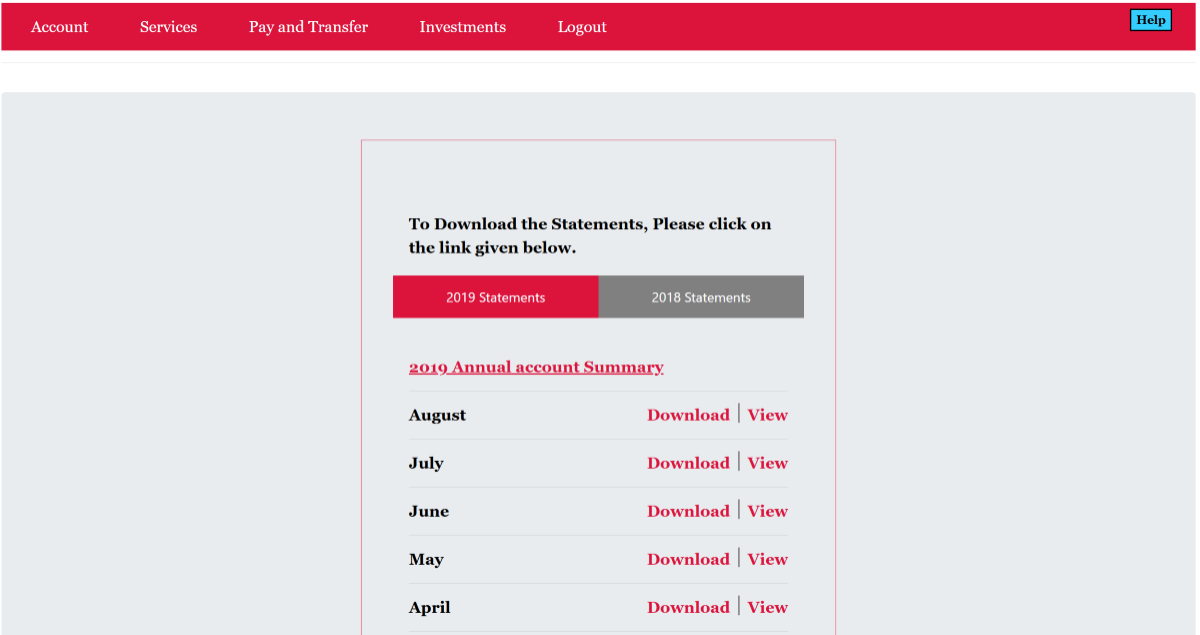
Screenshot of the view statements simulated web page.

The OMM Credit Card Task was the primary outcome variable. This task was developed iteratively, in collaboration with a computer scientist and other experts (Figure 1). As shown in Figure 2, the task consists of 2 broad areas (navigation and content) and 4 specific content-related aspects (simple and complex literacy, monitoring, and awareness). Screenshots of the webpages for this task are depicted in Figure 3. For the task, the participant first navigates 3 webpages (login page, accounts activity page, and view statements page; Figure 4) to access the credit card statement. The participants have to navigate these pages sequentially. They are able to progress to the next page (eg, from login to accounts activity page) only after they successfully enter the required information or click on the correct icon. Second, they answer 7 questions one at a time about the statement by clicking on the simulated pages (credit card basic literacy) and next identify erroneous transactions (statement monitoring). Third, they answer 9 questions about credit card management (complex credit card literacy) and, finally, rate their level of confidence about their performance before and after each task (self-awareness component). After completing the task, participant feedback about their perceived difficulty and comfort while performing this task is recorded. Performance metrics for this task include the number of clicks, accuracy, and total time to completion. Additional metrics are being developed and refined. Before each component of the task, participants are provided with practice to click on the responses or shown a demonstration as per the task requirements.

For participants enrolled via the ADRCs, cognitive data collected from those centers were going to be used to examine part of aim 1, which seeks to examine the validity of the task. In the ADRC, participants complete a standard battery of cognitive tests from the National Alzheimer's Coordinating Center Uniform Data Set Battery. Additional encompassing sociocognitive-emotional domains are outlined in Table 1. Moreover, a set of financial tasks was included in this study. The FCI-SF was adapted for online administration during the K99 phase, and 2 additional novel tasks and 2 online tasks, including the online ATM (automated teller machine) and banking tasks from the iFunction battery [41-43], were included in the R00 stage. Questions about experience with financial management and use of technology were completed via self-report, in which participants rated their confidence across various aspects of financial management, such as their everyday handling of finances, mathematical abilities, and use of an ATM.

**Table 1.** Select neuropsychological battery and key FDM^a^ measures.

Domain	Test
General cognitive ability	Montreal Cognitive Assessment–Alternate Form
Memory	NIHT^b^ Auditory Verbal Learning Test
Language or crystallized intelligence	NIHT Picture Vocabulary
Episodic memory	NIHT Picture Sequence Memory Test
Working memory	NIHT List Sorting Working Memory Test
Language or estimated intelligence	NIHT Oral Reading Recognition Test
Numeracy	Berlin Numeracy Scale
Practical judgment	Test of Practical Judgment
Financial capability^c^	Online ATM^d^ and banking task (iFunction)

a FDM: financial decision-making.

b NIHT: National Institutes of Health ToolBox Measure.

c Study-specific tests added to the battery.

d ATM: automatic teller machine.

A measure of financial exploitation was also incorporated during the R00 study phase. Financial exploitation is operationalized as financial loss due to identity theft, scam, or fraud that could have happened in person, or via phone, electronic, or online transaction. Participants completed a questionnaire about whether financial loss had ever occurred, the number of times it had occurred, and how it occurred (eg, theft, scam, fraud, repetitive spending, unnecessary purchases, etc). The survey was designed following the methodological recommendations of a recent meta-analysis, which found that multiple close-ended questions about specific fraud-scam events identified victims at a higher rate than studies using a single, general self-report question [44-46]. Preliminary data in people diagnosed with dementia indicates that 3 of 5 caregivers endorsed that financial loss had occurred in patients with instances ranging from 1 to 5 times. Of these 3 instances, 2 patients were endorsed by the caregivers as having “no concerns” about their ability to handle financial matters. For this project, a continuous, composite score ranging from 0 (never occurred) to n (number of times it occurred) will be used to quantify exploitation or loss for aim 4’s analysis.

### Recruitment and Screening Strategies

#### ADRC

The ADRC Data Core first ran a search query in its registry that fit our screening criteria. The ADRC recruitment coordinators or research staff conducted an initial screening. They referred participants to our study team once the ADRC staff made contact with an interested candidate and received consent to share their information with our study team. Additionally, during weekly ADRC consensus meetings, ADRC staff identified participants who fit our eligibility criteria and reached out to them for consent to share their information with us. This study’s staff contacted participants via phone or email to give study information and conduct further screening if the participant was interested. Consent and study procedures were conducted on the same day or at a later time point, depending on the participants' preferences. Individuals who expressed interest in this study were told the purpose of this study, the details of participation, and the potential risks and benefits before scheduling a study visit.

#### Saint Elizabeth’s Medical Center- Brighton/Boston, MA

Participants with and without cognitive impairment were to be recruited from Saint Elizabeth’s hospital in Brighton, MA. The PI provided Saint Elizabeth’s staff with Boston University’s IRB-approved and stamped flyers for them to give to patients. Interested individuals who contacted our study team were then screened, consented, and enrolled in this study. Participants recruited from Saint Elizabeth’s Medical Center were asked to sign a HIPAA (Health Insurance Portability and Accountability Act) release of medical records form so that the PI could access their medical records.

#### BMC-Boston, MA

Participants with and without cognitive impairment were to be recruited from BMC. BMC would identify and send out an initial opt-out message to individuals who may be eligible to participate in this study. The opt-out message was signed by the providers known to the patients recruited from BMC. Individuals were identified by their providers (during clinic visits) or by conducting a database search among the patients who received clinical care from neurologists or nurse practitioners using the criteria listed in the HIPAA section of our IRB. This study’s team then conducted screening and consented participants for this study. Participants recruited from BMC were asked to consent to link their data from BMC with the data collected in this study.

#### Community and Social Media

Healthy controls were recruited via social media platforms or from ResearchMatch with Boston University–stamped and approved posters. Interested individuals were contacted by this study’s team via phone or email. Individuals who expressed interest in this study were told the purpose of this proposed study, the details of participation, and the potential risks and benefits before obtaining their informed consent. We informed participants at the time of the prescreening phone call that we would do additional prescreening before consenting them to this study and that they may or may not be eligible at that time. The cognitive prescreener was administered to determine if a participant met the eligibility criteria.

### Data Collection and Monitoring

Participants consisted of older adults aged 60 to 75 years who are cognitively normal (Clinical Dementia Rating=0) as per the ADRC consensus conference. For participants recruited from the community, a Montreal Cognitive Assessment (MOCA) score of 26 or above was required for inclusion in this study. For those with a lower level of education, an education correction was applied to the total MOCA score. We focus on this age band to ensure that we are maximizing the probability of capturing older adults who use online services to manage their money. Participants complete this study with an examiner via a Zoom (Zoom Communications, Inc) call. The same set and order of tests are administered to all participants.

### Statistical Analysis

#### Overview

Primary covariates will include sex and years of education. For aims 1 and 2, we will conduct sensitivity analysis and then include variables such as socioeconomic status and financial literacy as secondary covariates to explore their influence on FDM. Secondary analysis will involve stratifying based on race and ethnicity and using these as covariates or as silent regressors.

#### Aim 1

For aim 1, we hypothesized that the OMM task would have high internal consistency, interrater, and test-retest reliability. Cronbach ω will be used to measure internal consistency reliability, Cohen κ for interrater reliability, and the Pearson product-moment correlation coefficient of stability for test-retest. Standard descriptive statistics of frequency, central tendency, and dispersion will be used to describe the sample. The NIH criteria of rigor will be incorporated when reporting the confirmatory factor analysis results, with emphasis on presenting information on the proposed models, modifications (if any) that were made, the specific measures that identify the latent variables, the intercorrelations among measures, the correlations between latent variables, and whether any constraints were used. When reporting the model fit statistics, the chi-square test, the root mean square error of approximation, the comparative fit index, and the standardized root mean square residual will be reported. A subgroup of participants has been retested on the same OMM credit card statement task after 3 months to evaluate test-retest reliability. The 2 raters will score the responses for the Complex Financial Literacy subcomponent following objectively defined criteria.

Similar to a previous study’s sample size [29], power analysis was conservatively conducted using α=.001. With n=200 (older adults; sex equally stratified), the minimum detectable effect size of *r*=0.20 at 80% power. A previous study, examining different FDM domains in a mixed group of older adults and those diagnosed with AD, reported strong correlations when assessing internal consistency (*r* ranged between 0.85 to 0.93; n=73), test-retest reliability (above 0.85, interval period=23 days, n=17), and high interrater reliability (n=11) [32]. Another study found money management to be associated with Dementia Rating Score (*r*=.80, *P*<.05; n=83) [32,47]. Thus, we have enough power to detect strong correlations.

#### Aim 2

For aim 2, we hypothesized that the rate of decline in statement monitoring and complex literacy would be associated with the rate of decline in executive functioning. Mixed effect models will be conducted with OMM task measures as dependent variables, time (2 levels: baseline and follow-up), domain (4 levels: basic financial literacy, complexity literacy, statement monitoring, and awareness), executive function scores, a 3-way interaction (time × domain × executive function) and their nested 2-way interactions as fixed effects controlling for age, gender, financial literacy, socioeconomic status, race, and education. The within-subject correlation is accounted for as a random intercept, and the correlation between domains will be specified as unstructured. We will first test the 3-way interaction term to test whether the change in executive function is differently associated with changes in OMM. If the 3-way interaction term is significant, we will run a contrast analysis to identify which domain changes show a significant association with changes in executive function. If the 3-way interaction term is not significant, we will rerun the mixed effect model without the 3-way interaction term and test the time × executive function. Power analyses were conducted with the assumption of moderate within-subject correlation (*r*=0.3) and conservatively estimating 20% attrition. With the sample size of 200, we expect to have at least 160 subjects at the end of this study. If the 2 domains show more than 0.38 SD decline (small effect size), we would be able to detect time × domain interaction with 80% power at the 5% significance level. If the association between change in executive function and change in complex literacy is greater than *r*=0.22, which is smaller than the medium effect size, we would have more than 80% power to detect such an association at the 5% significance level.

#### Aims 3 and 4

As the data from the AD sample could not be collected, aim 3 was not conducted, and aim 4 was modified. Originally, we had hypothesized that for aim 4, in those with clinical AD, compared to FDM as measured by OMM metrics (basic and complex literacy, and monitoring) and demographics (age, sex, and education), worse performance on OMM financial awareness will be strongly associated with a greater average number of times being financially exploited. Currently, we intend to examine the prevalence of financial loss in cognitively healthy older adults. Bivariate correlations will be conducted between demographics, FDM metrics (literacy and monitoring), financial awareness, and financial exploitation. Depending on the type of predictors, the three most significant correlations will be entered into a multiple linear regression analysis as predictors and the number of times financial exploitation occurred as the outcome variable. The mean difference in number of times financial exploitation occurred between groups for each predictor will be reported as β, along with 95% CIs. If the data are skewed or the range for the number of financial exploitation events is restricted, then multivariable logistic regression analysis will be conducted, with odds ratios and 95% CIs reported, to test whether each predictor is associated with financial exploitation as a dichotomous (yes or no) outcome. Separate models for each predictor will be constructed, along with a combined model that includes all 3 predictors included in order to assess whether each predictor is associated with financial exploitation independent of the other predictors. Models will be adjusted for age, sex, and education. Statistical significance will be based on a *P* value less than .05.

#### Power Analysis

For the primary hypothesis that lower financial awareness is associated with a higher likelihood of being financially exploited, power analysis was conducted under the assumption that the average proportion of having low financial awareness among those who reported being financially exploited is 1.54(p1), based on my preliminary data. With a sample size of 50 participants and assuming a 0.5 SD difference (ie, medium effect size) in p1 compared to p2, which represents the average proportion of having high financial awareness among those who report being financially exploited, we will have 83% power to detect such difference at the 5% significance level. This result implies that at our given sample size, we would have even greater power if we observe a larger difference (ie, greater than 0.5 SD difference). However, if the observed difference is smaller than 0.5 SDs, then we may be underpowered (ie, <80%) to detect such difference under our given sample size and assumptions.

## Results

Funding for the K99 and the R00 phases of the project was received on August 15, 2019, and January 1, 2021, respectively. This study was approved by the Columbia University Medical Center IRB on January 17, 2019, and by the Boston University IRB on March 30, 2022. Participant recruitment for the K99 phase began at Columbia University Medical Center in September 2019, while recruitment for the R00 phase began at Boston University in November 2022.

Enrollment concluded in July 2025. A total of 230 participants from the R00 group and 42 participants from the K99 group consented to this study, combining to a total of 272. Further, 147 participants returned to complete a longitudinal study visit. A subcohort of R00 participants (n=53) also completed a 3-month follow-up in which only the OMM task was completed. Preliminary results have been presented at various national and international conferences, including at the Gerontological Society of America’s meeting in 2020, and the International Neuropsychological Society’s meetings from 2023 to 2025 [48-50] and at the Gerontological Society of America’s meeting in 2020 [51]. Data analysis is underway as of August 2025, and results are expected to be published in 2026.

## Discussion

### Principal Findings and Implications

This proposed study was designed to use rigorous standards for developing the OMM credit card task. Study procedures were adapted during the K99 phase of this study. During the R00 phase, in sync with the K99 procedures, remote testing procedures were continued to maximize data and maintain standardization.

This proposed project has substantial implications for researchers, clinicians, and older adults. Digital currency is rapidly becoming the most preferred form of financial transaction. In fact, as per several studies such as the Pew Research Center’s data, the majority of older adults are internet savvy, with 78% of them being online and 77% owning a smartphone [52-54]. Development of the OMM task is highly relevant for the baby boomer cohort as well as the following generations and will reflect FDM abilities in a more appropriate and task-congruent manner. By objectively collecting FDM data in concert with comprehensive information about cognition, the proposed project will help researchers to expand their conceptualization of FDM and provide clinicians with a psychometrically sound, easy-to-administer, up-to-date assessment tool, ultimately helping older adults and their caregivers make informed determinations about managing their finances. Moreover, the simulation technology-based OMM task will provide new information that can be used to inform public health policies and perhaps banking and judicial policies, thereby preventing financial losses. Indeed, in a survey study of financial professionals, 74% compliance officers and 61% financial advisors reported encountering individuals with diminished capacity and feeling unprepared to handle the situation [55]. The availability of OMM will allow banks and judicial organizations to confidently develop roadmaps for handling cases related to questionable FDM and thereby create a balance between autonomy and safety.

### Potential Problems and Alternative Strategies

Challenges associated with confounding factors, low reliability, low validity, and attrition will be explored. We will attempt to report the rate of attrition along with the possible reasons (death, refusal to return, lost to follow-up, etc). A mixed-model statistical approach will be adopted to mitigate the effects of attrition. If we find specific patterns in attrition, the Inverse Probability of Treatment Weighting approach will be used to adjust for differential attrition rates [56]. There is a possibility that no change will be detected in older adults at longitudinal follow-up. If changes are detected, then the OMM task can serve as a sensitive test for detecting functional impairments in preclinical MCI. If, however, no changes are detected, then the data will serve as a useful reference for comparing other clinical groups and for deriving normative data.

### Dissemination Plan

Once the task is validated, a training manual with administration instructions, scoring, and interpretation procedures, along with a demonstration video of the OMM task, will be developed to enable standardization of the OMM application. Online training will be provided to researchers and clinicians on an annual basis as per institutional guidelines. We anticipate peer-reviewed publication of our findings after 2025. Findings will be shared at national and international conferences via oral talks and poster presentations. Deidentified data will be provided upon request by the researcher and as per journal and institutional policies to encourage reproducibility across diverse settings.

### Future Directions

The findings from this study will be used to build several closely related projects. First, for the next iteration of the OMM task, the plan is to automate the task such that participants can complete the task remotely without the involvement of research staff. The results will be automatically generated, and if the researcher or clinician needs to view the task performance, they will be able to review the performance via automatically recorded videos. Next, the OMM task needs to be validated in clinical and demographically diverse populations. Similarly, the links between this task and individuals with varying types and levels of biomarker statuses need to be examined. Third, the neural basis of the OMM task should be evaluated as this might have implications for diagnosis, monitoring, and rehabilitation.

## Supplementary material

10.2196/82488 Peer Review Report 1 Peer review report by Pathway to Independence Award Committee (National Institutes of Health, United States).

## Abbreviations

AD: Alzheimer disease
ADRC: Alzheimer’s Disease Research Center
ATM: automated teller machine
BMC: Boston Medical Center
COSMIN: Consensus-Based Standards for the Selection of Health Measurement Instruments
FCI-SF: Financial Capacity Instrument – Short Form
FDM: financial decision-making
HIPAA: Health Insurance Portability and Accountability Act
IRB: institutional review board
MOCA: Montreal Cognitive Assessment
NIH: National Institutes of Health
OMM: Online Money Management
PI: principal investigator
