# Supplementary material for: Objective Assessment of Financial Decision-Making With a Simulated Online Money Management Task in Older Adults: Protocol for a Prospective Observational Study
Source: JMIR Res Protoc. 2026 Apr 20;15:e82488. doi: 10.2196/82488 (PMC13094792; doi:10.2196/82488)
Supplement: Peer Review Report 1 [file resprot-v15-e82488-s001.pdf]

RESUME AND SUMMARY OF DISCUSSION: This is an application, NIH Pathway to Independence Award (K99/R00), from Columbia University (CU), on behalf of the Principal Investigator (PI), Dr. Preeti Sunderaraman. It requests five years of funding to support the candidate's goal of modernizing the assessment of financial decision making. Specifically, the application proposes to develop and evaluate the reliability, validity and feasibility of a novel simulated-technology based online money management (OMM) task in a cognitively diverse sample of older adults. Dr. Sunderaraman is a strong candidate, with a Ph.D. in neuropsychology from Drexel University (DU). The candidate is currently a Postdoctoral Fellow at CU with 7 first authored publications. Dr. Sunderaraman has an excellent mentoring team consisting of Drs. Cosentino and Yaakov Stern and a strong advisory committee both with complementary areas of expertise. The training plan includes both formal and informal coursework in machine learning, latent variable methods, advanced psychometrics, and use of the Health and Retirement Study (HRS). Additional seminars, hand-on training and seminar/conference attendance encompass the training plan. Excellent references letters are provided attesting to Dr. Sunderaraman's competency and experience. The career development plan described bodes well towards leading Dr. Sunderaraman into an independent research career. Concerns target the methodological components of the application, such as, while the proposed analyses will take into account demographics there is no information about more proximal confounders such as the subject's prior history of using the internet for financial related tasks and even whether they utilize credit cards. The application lacks a discussion on the ownership of the intellectual property rights attached to the simulation task implementation. The online money management (OMM) program, while important, may be too narrow in terms of skill-building for an independent research career. The described weaknesses diminished the enthusiasm somewhat for this application.

DESCRIPTION (provided by applicant): The advent of digital finance and online money management (OMM) services has changed the ways in which we conduct everyday transactions. Based on my preliminary F32 survey findings, the number of older adults who use technology is drastically growing and the way they approach financial transactions is certainly changing. Given the increasing number of older adults and corresponding diseases of aging such as Alzheimer's Disease (AD), the vulnerability of older adults to financial exploitation, and the pervasiveness of financial decision making in everyday life, it is critical to be able to accurately examine financial decision-making using objective, quantifiable, and ecologically valid tasks that are in keeping with the current trends. In 2016, an expert panel working for the US Social Security Administration found a gap between the current assessment of financial decision making and real-world financial performance. The current proposal seeks to bridge this gap by developing and applying simulation technology-based OMM task to assess financial decision making in individuals across the spectrum of cognitive aging. Accordingly, the first two aims (K99/Mentored phase) of the current proposal are to establish the reliability and validity of a novel, simulated OMM credit card statement review task in cognitively healthy older adults via psychometrically rigorous approaches. The third and fourth inferential aims (R00 phase) are to use: (i) a longitudinal strategy to track OMM over time and in relation to cognitive changes in cognitively healthy older adults, and (ii) a cross-sectional strategy to examine OMM in two clinical groups (mild AD and those judged as financially incapable by Adult Protective Services) versus healthy older adults. To accomplish these aims, I will develop advanced skills to supplement my strong neuropsychological training, by: 1) gaining expertise in developing and conducting functional assessments using state-of-the-art technology; 2) developing a deeper knowledge of psychometric measurement to optimize task design; and 3) enriching my conceptual understanding of financial decision making while learning how to incorporate neuroeconomic principles and metacognitive assessment in financial decision making measurement. I have assembled an excellent and well-rounded mentorship team consisting of a

computer scientist, biostatistician, gerontologist, economist, and expert neuropsychologists. Overall, this K99/R00 proposal enables me to lay a foundation for an independent research career focused on using technology to accurately conceptualize financial decision making in cognitively diverse older adults, a goal that is in line with the NIA's mission to improve "the health, well-being, and independence of adults as they age".

PUBLIC HEALTH RELEVANCE: Older adults, a wealthy segment of the population, are known to exhibit compromised financial decision making especially in the context of age-related diseases such as Alzheimer's Disease. As the landscape of financial decision making has changed dramatically with rapid technological advances, there is a growing gap between the skills necessary for everyday financial decision making and the way it in which it is assessed clinically. Given the precipitous, ongoing and projected increase in the number of older adults and their use of technology, it is imperative to develop financial decision making measures that approximate real world functioning in the current technological culture that can inform clinical determination of financial decision making abilities, and shape public policies aimed at minimizing financial losses.

CRITIQUE 1:

Candidate: 2
Career Development Plan/Career Goals/Plan to Provide Mentoring: 2
Research Plan: 2
Mentor(s), Co-Mentor(s), Consultant(s), Collaborator(s): 2
Environment, Commitment to the Candidate: 1

Overall Impact:
This outstanding candidate is a PhD trained neuropsychologist who is currently doing a postdoc with an F32 which she secured for the work. All letters by mentors and references offer a high degree of praise for her capability to conduct independent research and move to independence. Through her doctoral and postdoc work she has been focused on the issues of the assessment of financial decision-making capacity. Recognizing that individuals, including older adults, currently conduct financial transactions using electronic resources and that this is not part of most current instruments, she proposes to develop an electronic performance task based on online credit card statements which she then will evaluate in a cognitively normal cohort for reliability and other psychometric properties during the K99 phase. During the proposed R00 portion she will evaluate the validity of the measure by studying longitudinal changes and differences between clinically defined groups. Despite some methodologic concerns, high enthusiasm is expressed for this application.

1. Candidate:
Strengths
- Dr. Sunderaraman is a PhD trained Neuropsychologist (Drexel) who is currently being supported by an F32 which she obtained for postdoctoral work at Columbia University.
- All letters by mentors and references offer their highest degree of support for her capability to conduct independent research and develop an ongoing research program
- She is also described as a talented clinician who expresses sincere concern for her patients and is accomplished in neuropsychological assessment.
- Her current focus is on developing methods to assess financial decision making in older adults and neurologically vulnerable groups.

- Her dissertation aimed to investigate the influence of neurocognitive abilities to financial capacity in individuals with chronic acquired brain injury and her F32 is focused on financial decision making in young and older adults.
- She has published 7 first authored journal articles.

Weaknesses
- Not provided.

2. Career Development Plan/Career Goals & Objectives/Plan to Provide Mentoring:
Strengths
- The Mentored (K99) phase is to implement a computerized credit card report in order to measure the ability of the subject to correctly navigate and consume the financial information. This results of the simulation task will then be scored to provide a standardized assessment.
- After Learning about necessary psychometric concepts, she will first evaluate the psychometric properties of the simulation task in an initial cohort of 200 healthy older adults. This will include estimates of both reliability and validity.
- Her overarching goal is to lay the foundation for a career focused on developing and implementing comprehensive, ecologically valid, technology-based assessment methods for everyday financial decision making. The K99 portion contains necessary didactic and one on one interactions to support this.
- Columbia offers access to an array of appropriate seminars, rounds and other relevant experiences.
- As is customary for the T32 program in the Cognitive Neuroscience Division, an Individual Development Plan will be tailored which will contain appropriate milestones which will be reviewed by her mentors and advisors.

Weaknesses
- While she is being mentored by an EE faculty and they have been collaborating since early this year, it is not clear what the platform is that is required to support this simulation (e.g. is it web based, run on a laptop or an app on a tablet?)

3. Research Plan:
Strengths
- Well-developed strategy for initial studies to determine longitudinal characteristics in a normal population and for its ability to discriminate between groups based on their dementia diagnosis and their financial competencies.
- Both the longitudinal studies and the clinical groups will provide excellent pilot data for the design of R01 proposals to continue this line of research in the institution which offers a tenure track appointment for the R00 phase.
- Appropriate attention attached to rigor and reproducibility criteria and sex effects will be explored.

Weaknesses
- It appears that during Aim 4, separate recruitment will be done for clinical groups, thus reducing the possibility for masking to reduce bias by those administering the simulation tasks.

4. Mentor(s), Co-Mentor(s), Consultant(s), Collaborator(s):
Strengths
- Dr. Stephanie Cosentino, the Primary mentor is a neuropsychologist focused on metacognition and cognitive aging and the applicant's current postdoctoral mentor. She has support on a number of relevant NIH grants. While Dr. Cosentino she is relatively

junior as a K-award mentor, she is supported by her mentor, Dr. Yaakov Stern, who has a long history of supervising developing researchers.
- Additional mentors and advisors include an appropriate array of expertise including Dr. Melanie Wall in Biostatistics, Dr. Peter Lichtenberg, a world renown leader in creating person-centered financial decision making scales, Dr. Teresa Ghilarducci in retirement planning, Dr. Jason Krellman an expert in conducting neuropsychology assessments, Dr. Tania Giovannetti an expert in investigating everyday function and Dr. Ziqian Dong, an Electrical Engineer (EE). An appropriate schedule of meetings with these mentors/advisors is proposed.

Weaknesses
- Not provided.

5. Environment and Institutional Commitment to the Candidate:
Strengths
- Columbia is an outstanding environment for this candidate to conduct the K99 portion and she will be imbedded in an outstanding intellectual environment.

Weaknesses
- None noted.

Protections for Human Subjects:
Acceptable Risks and Adequate Protections.

Data and Safety Monitoring Plan:
Acceptable.

Inclusion of Women, Minorities, and Children:
- Sex/Gender: Distribution justified scientifically.
- Race/Ethnicity: Distribution justified scientifically.
- Inclusion/Exclusion of Children under 18: Excluding ages <18; justified scientifically.

Training in the Responsible Conduct of Research:
Acceptable.
Comments on Format:
- Acceptable.

Comments on Subject Matter:
- Acceptable.

Comments on Faculty Participation:
- Acceptable.

Comments on Duration:
- Acceptable.

Comments on Frequency:
- Acceptable.

Resource Sharing Plans:
Acceptable.

Budget and Period of Support:
Recommend as Requested.

CRITIQUE 2:

Candidate: 1
Career Development Plan/Career Goals/Plan to Provide Mentoring: 2
Research Plan: 3
Mentor(s), Co-Mentor(s), Consultant(s), Collaborator(s): 1
Environment, Commitment to the Candidate: 1

Overall Impact:
This K99 request funding for Dr. Preeti Sunderaraman, a Postdoctoral Fellow to receive training in financial decision making and methods. The candidate is very strong, having had an F32 and already developing the online money management assessment tool. The research is in an important area and the mentor team is expert. The research plan is ambitious and requires more clarity in recruitment capacity and criteria. Training plan relies too much on online learning.

1. Candidate:
Strengths
- PhD in neuropsychology from Drexel.
- Postdoctoral fellow at Columbia.
- NIH NRSA F32 Postdoctoral Fellowship in Neuropsychology.
- 11 publications, including 4 first author in PubMed.

Weaknesses
- None noted.

2. Career Development Plan/Career Goals & Objectives/Plan to Provide Mentoring:
Strengths
- Coursework in machine learning, latent variable modeling, etc. is extensive.

Weaknesses
- It would help to group activities by goals.

3. Research Plan:
Strengths
- Financial decision making is an important topic.
- Online money management (OMM) simulated task would be an important tool.
- Analyses models are detailed.

Weaknesses
- Exclusions MOCA score <= 26 may be too high, especially among non-whites and lower educated (even with adjustment for education). This will limit you potential cognitive range for testing.
- MOCA<=26 is mild MCI, not necessarily Mild dementia.
- It is unclear why maximum age of 75
- It is unclear how non-impaired is derived from the Alzheimer's Disease Research Center.
- It is unclear how long is OMM. The rest of the battery is quite extensive.
- It is unclear how many people the investigative team will be able to sample from these sources and will the sample be sufficient.

4. Mentor(s), Co-Mentor(s), Consultant(s), Collaborator(s):
Strengths

- Dr. Stephanie Cosentino is an Associate Professor of Neuropsychology. This is first K mentorship, but has 3 R01s and 2 U-mechanism grants.
- Dr. Peter Lichtenberg is leader in financial decision-making scales and has extensive research and has mentoring experience.
- Dr. Cecelia Dong has National Science Foundation grant.
- Dr. Tania Giovannetti has 49 publications and diverse funding.
- Dr. Melanie Wall is biostatistician with over 300 publications and involved in numerous grants.
- Other advisors have good background.

Weaknesses
- None noted.

5. Environment and Institutional Commitment to the Candidate:
Strengths
- Columbia University has strong environment.
- Letter of commitment clarifies protected time.

Weaknesses
- None noted.

Protections for Human Subjects:
Acceptable Risks and Adequate Protections.
- Detailed plan, some concern about length of assessment as burdensome.

Data and Safety Monitoring Plan:
Acceptable.

Inclusion of Women, Minorities and Children:
- Sex/Gender: Distribution justified scientifically.
- Race/Ethnicity: Distribution justified scientifically.
- Inclusion/Exclusion of Children under 18: Excluding ages <18; justified scientifically.
- Gender discussed. Minorities not discussed but included in sample.

Training in the Responsible Conduct of Research:
Acceptable.
Comments on Format:
- Acceptable. Formal courses, online trainings, seminar discussions, mentor meetings.

Comments on Subject Matter:
- Acceptable. Comprehensive.

Comments on Faculty Participation:
- Acceptable. Weekly.

Comments on Duration:
- Adequate.

Comments on Frequency:
- Adequate.

Resource Sharing Plans:
Acceptable.
- Data-sharing agreement.

Budget and Period of Support:
Recommend as Requested.

CRITIQUE 3:

Candidate: 1
Career Development Plan/Career Goals/Plan to Provide Mentoring: 2
Research Plan: 2
Mentor(s), Co-Mentor(s), Consultant(s), Collaborator(s): 1
Environment, Commitment to the Candidate: 1

Overall Impact:
This is a K99 application for an NIH Pathway to Independence Award for Dr. Preeti Sunderaraman, a Postdoctoral Fellow in Neuropsychology, at Columbia University. She plans to use this award to become an" independent, innovative and dedicated research scientist in the field of financial decision making with the goal of augmenting our conceptualization of this construct and by developing rigorous and ecologically valid assessments to inform public policies". The goal to gain skills in financial decision-making is unique in which Dr. Sundararaman could develop a useful niche. She has been involved in a very productive career in both India and the US as a clinical psychologist. Her dissertation led to work in the field of decision-making. competency which led the way to her current goals. Her post-doctoral research has provided experience in cognitive abilities (crystallized and fluid), brain structures, and affective states that correlate with financial decision making in older adults, an understudied field. An evaluation of her progress assures Dr. Sunderarama will stay on target for her goals. Aims of her study primarily involve online money management (OMM) which may be a bit narrow in terms of skill-building. A scientific premise for all aims is provided. Use of a financial capability instrument along with neuropsychiatric testing provides excellent data for her thesis.

1. Candidate:
Strengths
- Dr. Sunderaraman has had a very productive career in both India and the US as a clinical psychologist.
- Her dissertation led to work in the field of decision-making. competency which led the way to her current goals.
- Her post-doctoral research has provided experience in cognitive abilities which is also relevant for this training and proposal.

Weaknesses
- None noted.

2. Career Development Plan/Career Goals & Objectives/Plan to Provide Mentoring:
Strengths
- The training plan includes both formal and informal training.
- Courses in machine learning, latent variable methods, advanced psychometrics are applicable and important.
- Learning to use the data in the Health and Retirement Study (HRS) is very valuable.
- Additional seminars and experiential learning enhances the plan.

Weaknesses
- Not provided.

3. Research Plan:
Strengths
- The goal to gain skills in financial decision-making is unique in which Dr. Sundararaman could develop a useful niche.
- This is an understudied field of research.
- The need to help older adults with financial decision-making is significant.

Weaknesses
- Not provided.

4. Mentor(s), Co-Mentor(s), Consultant(s), Collaborator(s):
Strengths
- The mentoring team is excellent, and skills complement each other.

Weaknesses
- None noted.

5. Environment and Institutional Commitment to the Candidate:
Strengths
- Very good.

Weaknesses
- None noted.

Protections for Human Subjects:
Acceptable Risks and Adequate Protections.

Data and Safety Monitoring Plan:
Acceptable.

Inclusion of Women, Minorities, and Children:
- Sex/Gender: Distribution justified scientifically.
- Race/Ethnicity: Distribution justified scientifically.
- Inclusion/Exclusion of Children under 18: Excluding ages <18; justified scientifically.

Training in the Responsible Conduct of Research:
Acceptable.
Comments on Format:
- Acceptable.

Comments on Subject Matter:
- Acceptable.

Comments on Faculty Participation:
- Acceptable.

Comments on Duration:
- Acceptable.

Comments on Frequency:
- Acceptable.

Resource Sharing Plans:
Acceptable.

Budget and Period of Support:
Recommend as Requested.

THE FOLLOWING SECTIONS WERE PREPARED BY THE SCIENTIFIC REVIEW OFFICER TO SUMMARIZE THE OUTCOME OF DISCUSSIONS OF THE REVIEW COMMITTEE, OR REVIEWERS' WRITTEN CRITIQUES, ON THE FOLLOWING ISSUES:

PROTECTION OF HUMAN SUBJECTS: ACCEPTABLE. Acceptable risks and/or adequate protections are detailed in the application.

INCLUSION OF WOMEN PLAN: ACCEPTABLE. The investigative team will try to enroll an equal number of males and females. If, however, enrollment becomes imbalanced, every effort will be made to increase the enrollment of the
underrepresented gender.

INCLUSION OF MINORITIES PLAN: ACCEPTABLE. No potential participant will be excluded based on gender/sex, race, or ethnicity.

INCLUSION OF CHILDREN PLAN: ACCEPTABLE. Children are excluded from this research study.

COMMITTEE BUDGET RECOMMENDATIONS: The budget was recommended as requested.

Footnotes for 1 K99 AG062783-01; PI Name: Sunderaraman, Preeti

NIH has modified its policy regarding the receipt of resubmissions (amended applications). See Guide Notice NOT-OD-14-074 at http://grants.nih.gov/grants/guide/notice-files/NOT-OD-14-074.html. The impact/priority score is calculated after discussion of an application by averaging the overall scores (1-9) given by all voting reviewers on the committee and multiplying by 10. The criterion scores are submitted prior to the meeting by the individual reviewers assigned to an application, and are not discussed specifically at the review meeting or calculated into the overall impact score. Some applications also receive a percentile ranking. For details on the review process, see
http://grants.nih.gov/grants/peer_review_process.htm#scoring.
